# Average Information Content Maximization—A New Approach for Fingerprint Hybridization and Reduction

**DOI:** 10.1371/journal.pone.0146666

**Published:** 2016-01-19

**Authors:** Marek Śmieja, Dawid Warszycki

**Affiliations:** 1 Faculty of Mathematics and Computer Science, Jagiellonian University, 6 Lojasiewicza Street, 30-348 Kraków, Poland; 2 Institute of Pharmacology, Polish Academy of Sciences, 12 Smetna Street, 31-343 Kraków, Poland; University of Edinburgh, UNITED KINGDOM

## Abstract

Fingerprints, bit representations of compound chemical structure, have been widely used in cheminformatics for many years. Although fingerprints with the highest resolution display satisfactory performance in virtual screening campaigns, the presence of a relatively high number of irrelevant bits introduces noise into data and makes their application more time-consuming. In this study, we present a new method of hybrid reduced fingerprint construction, the Average Information Content Maximization algorithm (AIC-Max algorithm), which selects the most informative bits from a collection of fingerprints. This methodology, applied to the ligands of five cognate serotonin receptors (5-HT_2*A*_, 5-HT_2*B*_, 5-HT_2*C*_, 5-HT_5*A*_, 5-HT_6_), proved that 100 bits selected from four non-hashed fingerprints reflect almost all structural information required for a successful in silico discrimination test. A classification experiment indicated that a reduced representation is able to achieve even slightly better performance than the state-of-the-art 10-times-longer fingerprints and in a significantly shorter time.

## Introduction

**Fig 1:**
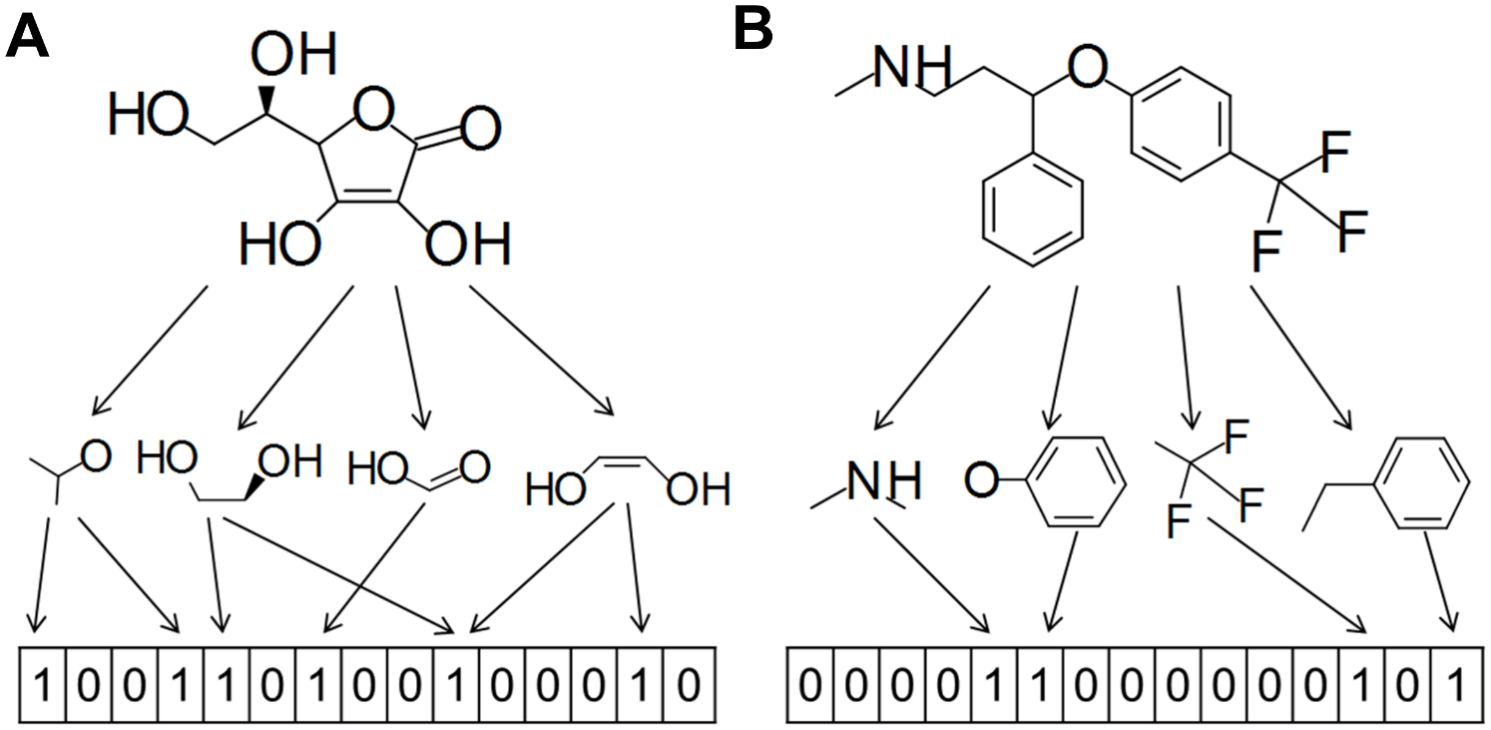
Exemplary hashed (A) and non-hashed (B) fingerprints. Presence of “1” and “0” corresponds to presence or absence of a particular pattern, repectively. In case of hashed fingerprint (A) bit collision phenomena is presented—one bit encodes more than one motif.

Fingerprints are one of the most popular methods of converting chemical structures into a form that can be used in, e.g., machine learning experiments. They encode a compound’s structural features into a bitstring, where “1” and “0” mean the presence or absence, respectively, of a particular pattern. Fingerprints are divided into two subgroups: non-hashed fingerprints (e.g., Substructure fingerprint, Klekotha-Roth fingerprint), which encodes precisely defined structural patterns, and hashed fingerprints (e.g., Extended fingerprint, Graph-only fingerprint) which are without an assigned meaning for each bit (Fig 1). Fingerprints are widely used in classification problems or similarity searching; therefore, they have found application in computer-aided drug design campaigns [1–8].

A multitude of structural features present in chemical compounds results in fingerprints, among which, the longest one contains 4860 bits [9]. The physical impossibility of the occurrence of hundreds of chemical substructures in low-molecular-weight chemical compounds and the biological insignificance of many bits increase the noise level in classification experiments. Moreover, the high resolution of the data increases the computational time, which is crucial in large virtual screening cascades.

Therefore, the reduction of fingerprint length without the loss of any meaningful information has become an important cheminformatics challenge in recent years. Several methodologies, e.g., consensus fingerprints [10], bit scaling [11], reverse fingerprints [12] and bit silencing [13] were introduced to reduce fingerprints via the weighting of particular bits. Another approach proposed by Nisius et al. selects fingerprint bits according to their discrimination power which is measured by Kullback-Leibler divergence [14]. The method was applied to single fingerprints as well as to collections of fingerprints, leading to a successful attempt at fingerprint hybridization. [15].

In this study, we introduce a new method for fingerprint hybridization and reduction—Average Information Content Maximization (AIC-Max algorithm). The algorithm uses an extended version of mutual information, hereafter referred as the Average Information Content (AIC), to select the most informative bits of different fingerprints needed for splitting active from inactive compounds. In contrast to the aforementioned techniques, the AIC-Max algorithm may construct an optimal fingerprint for several biological targets. This approach substantially extends its application area. The strength of the AIC-Max algorithm stems from the fact that the selection process evaluates the discrimination power of entire groups of bits instead of single ones. Consequently, the algorithm will not select two features that carry similar information.

**Fig 2:**
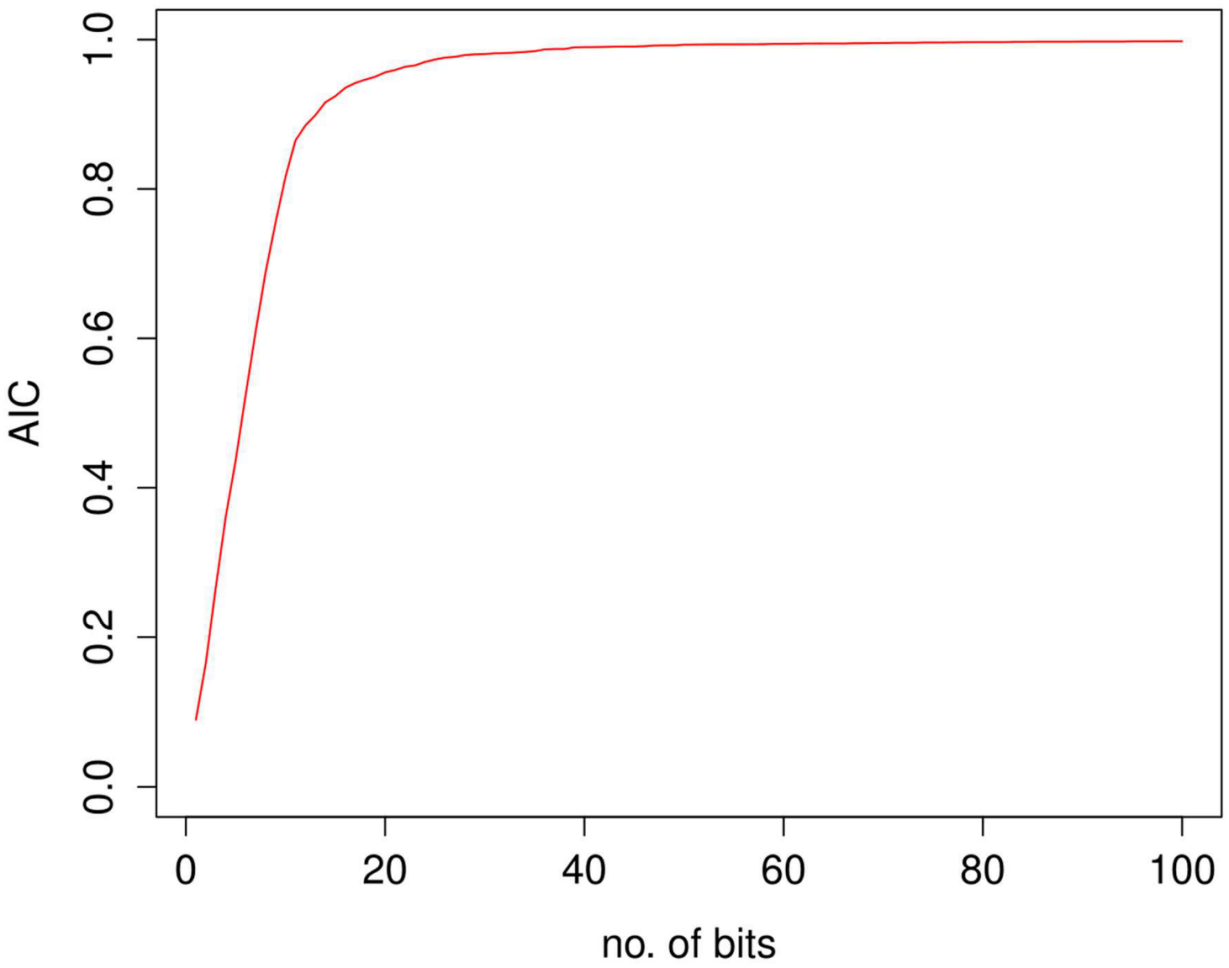
The relationship between the number of bits selected by the AIC-Max algorithm and information related activity. The information, measured by AIC Eq (1), was averaged over all datasets used in the underlying study.

The proposed methodology was applied to create a reduced representation dedicated to the analysis of five closely related serotonin receptors: 5-HT_2*A*_, 5-HT_2*B*_, 5-HT_2*C*_, 5-HT_5*A*_ and 5-HT_6_ (members of the G-protein coupled receptor superfamily) that play an important role in, e.g., the central nervous system (CNS) [16]. The algorithm was additionally tested on four other targets families: carbonic anhydrases, cathepsins, histamine receptors and kinases (See S1 File). Although the advantages of hashed fingerprints cannot be denied, only non-hashed fingerprints were considered in the current study. This conscious abandonment of hashed fingerprints was due to the lack of predefined substructural features and bit collision phenomenon (the same bit is set by multiple patterns) commonly occurring in those fingerprints [17], which make the structural interpretation of particular fingerprint coordinates nearly impossible. A hybrid fingerprint, reduced to 100 bits, reflects 99.77% of the information needed to distinguish active compounds from inactive ones (Fig 2) and contains structural patterns typical for serotonin receptors ligands, such as positively polarizable nitrogen atoms and aromatic systems.

A reduced representation significantly outperformed four standard non-hashed fingerprints in a classification experiment and achieved slightly better results in comparison to hashed fingerprints generated by PaDEL software [18] when a random forest classifier [19] was used. Moreover, the average training time of the random forest predictor compared to the Extended fingerprint was reduced almost 20 times. The constructed fingerprint generalized well to related biological targets such as the 5-HT_1*A*_ receptor as shown by additional tests. The results indicate that AIC-Max algorithm is an efficient method for fingerprint reduction and hybridization, opening new perspectives for both virtual screening campaigns and structural analysis of chemical space covered by ligands acting on similar targets.

## Materials and Methods

The Average Information Content Maximization algorithm (AIC-Max algorithm) uses the notion of Average Information Content (AIC) to rank the features by their significance. The AIC quantifies the percentage of information that a set of features $X=\{X_{1},\ldots ,X_{N}\}$ carries of the activity with respect to a set of biological receptors R={1,…,K} (the corresponding set of activity variables will be denoted by $Y=\{Y_{1},\ldots ,Y_{K}\}$). The AIC is defined as the mutual information $\text{MI}(X;Y_{i})$ normalized by the entropy SE(*Y*_*i*_) [20–22], averaged over R

$$\begin{array}{ccc} {\text{AIC}}_{Y}\left(X\right) & = & \frac{1}{K}\sum_{i=1}^{K}\frac{\text{MI}(X;Y_{i})}{\text{SE}(Y_{i})}\\ & = & \frac{1}{K}\sum_{i=1}^{K}\frac{\sum_{x\in S_{N}}\sum_{y\in \{0,1\}}P_{i}\left(x;y\right)\log_{2}\frac{P_{i}\left(x;y\right)}{P\left(x\right)P_{i}\left(y\right)}}{-\sum_{y\in \{0,1\}}P_{i}\left(y\right)\log_{2}P_{i}\left(y\right)}, \end{array}$$ (1)

where *S*_*N*_ = {0,1}^*N*^ is a set of all binary sequences of length *N* and *P*_*i*_(*y*), *P*(*x*), *P*_*i*_(*x*;*y*) denote the probabilities that {*Y*_*i*_ = *y*}, {*X*_1_ = *x*_1_, …, *X*_*N*_ = *x*_*N*_}, {*X*_1_ = *x*_1_, …, *X*_*N*_ = *x*_*N*_, *Y*_*i*_ = *y*}, respectively.

If X fully determines the activity of all receptors, then AIC = 1; for X independent of all elements of Y, it returns value 0. The set of features that reflects all the information of the activity against *l* receptors and none of the information for the remaining (*k* − *l*) receptors gives $\text{AIC}=\frac{l}{k}$, as demonstrated in Table 1. For closely related biological targets, however, the most informative features usually overlap to a large extent.

**Table 1:** Minimal and maximal values of AIC. The 3-bit fingerprint representation *X*_1_ *X*_2_ *X*_3_ of eight compounds and their activity labels *Y*_1_, *Y*_2_, *Y*_3_ given three biological targets, as listed in the table. Since the activity of the *i*-th receptor is fully determined by a single feature *X*_*i*_, then AIC_*Y*_*i*__(*X*_*i*_) = 1, for *i* = 1,2,3. In contrast, AIC_*Y*_*i*__(*X*_*j*_) = 0, for *i* ≠ *j* because *Y*_*i*_ is independent of *X*_*j*_. Finally, ${\text{AIC}}_{\left\{Y_{1},Y_{2},Y_{3}\right\}}\left(X_{1},X_{2}\right)=\frac{2}{3}$, since the activity of two out of three receptors was fully reflected by two bits.

compound no.	*X*_1_	*X*_2_	*X*_3_	*Y*_1_ = *X*_1_	*Y*_2_ = *X*_2_	*Y*_3_ = *X*_3_
1	0	0	0	0	0	0
2	0	0	1	0	0	1
3	0	1	0	0	1	0
4	0	1	1	0	1	1
5	1	0	0	1	0	0
6	1	0	1	1	0	1
7	1	1	0	1	1	0
8	1	1	1	1	1	1

The important point is that the value of AIC depends on the joint information contained in all features included in X. In particular, if *X*_1_ = *X*_2_ then

$$\begin{array}{c} {\text{AIC}}_{Y}\left(X_{1},X_{2}\right)={\text{AIC}}_{Y}\left(X_{1}\right)={\text{AIC}}_{Y}\left(X_{2}\right). \end{array}$$

The above equality always holds if the correlation between *X*_1_ and *X*_2_ equals 1. In other words, the repeated addition of the same feature does not increase the value of AIC. In contrast, the extension of the set of features by an additional element cannot decrease AIC, as illustrated in Table 2.

**Table 2:** Influence of dependent and independent bits on AIC. The activity of a given receptor depends only on two out of four features: *X*_1_ and *X*_2_. The addition of feature *X*_3_ to *X*_1_ does not change AIC because it is independent of *Y*, which results in AIC_*Y*_(*X*_1_) = AIC_*Y*_(*X*_1_, *X*_3_) = 0.38. The same holds for *X*_4_, which is completely correlated with *X*_1_, and AIC_*Y*_(*X*_1_) = AIC_*Y*_(*X*_1_, *X*_4_) = 0.38.

compound no.	*X*_1_	*X*_2_	*X*_3_	*X*_4_ = NOT(*X*_1_)	*Y* = *X*_1_ AND *X*_2_
1	0	0	0	1	0
2	0	0	1	1	0
3	0	1	0	1	0
4	0	1	1	1	0
5	1	0	0	0	0
6	1	0	1	0	0
7	1	1	0	0	1
8	1	1	1	0	1

To calculate AIC for a given set of receptors R, the datasets of compounds for each r∈R can be created separately. This consideration implies that a single instance (compound) does not have a known activity label for all considered receptors. It is an important property because most of the compounds have proven activity (or inactivity) only for one receptor. It is worth mentioning that this reasoning cannot be applied to classical mutual information, where the activity of every compound has to be provided to perform analogical evaluation.

Given a set F of all features (fingerprint coordinates), the goal is to find an *N*-element subset X of F such that ${\text{AIC}}_{Y}\left(X\right)$ is maximal. In practice, it might be impossible to calculate AIC for all subsets of features to determine the most informative one (e.g, the number of *m*-element subsets of *n*-features equals (nm) which even for *n* = 1000 and *m* = 10 gives about 2 ⋅ 10^23^). The proposed AIC-Max algorithm uses a heuristic search in the space of all features F to reduce the computational time of the entire selection process. It iteratively picks these coordinates X∈F∖X which maximize ${\text{AIC}}_{Y}\left(X\cup \left\{X\right\}\right)$—the information contained in already chosen features. The selection of *N* features is described as follows:

AIC-Max algorithm:

Input: F – set of given features

 Output: X – set of selected features

 1. initialize X=∅,

 2. iterate *N*-times:

  (a) find X∈F∖X which maximizes ${\text{AIC}}_{Y}\left(X\cup \left\{X\right\}\right)$,

  (b) update X=X∪{X}.

To provide more efficient computations, the calculation of AIC in step 2a can be performed for a randomly selected *n* ≤ *N* element subset of X—in the experiments we used *n* = 10.

The concept of the AIC is based on information theory and is partially related to Asymmetric Clustering Index [23]. The most fundamental concept in information theory is Shannon entropy (SE), which quantifies the information contained in a given feature *X* [20]. Formally, if *X* takes values in {1, …, *k*}, then:

$$\begin{array}{c} \text{SE}\left(X\right)=-\sum_{i=1}^{k}P\left(i\right)\log_{2}P\left(i\right), \end{array}$$

where *P*(*i*) is a probability of observation {*Y* = *i*}. Note, that SE(*Y*) = 0 if *X* = *constant*. In contrast, if all values of *X* are equally probable, then SE attains a maximal value of log_2_ *k*.

To measure the joint information shared by two features, the notion of mutual information (MI) has to be used [20]. For *X* and *Y* taking values in {1, …, *k*}, the MI is formulated as follows:

$$\begin{array}{c} \text{MI}\left(X;Y\right)=\sum_{i=1}^{k}\sum_{j=1}^{k}P\left(i;j\right)\log_{2}\frac{P(i;j)}{P(i)P(j)}, \end{array}$$ (2)

where *P*(*i*;*j*) is the probability that {*X* = *i*, *Y* = *j*}. It can also be naturally extended to the set of features $X=\left(X_{1},\ldots ,X_{n}\right),Y=\left(Y_{1},\ldots ,Y_{k}\right)$: the indexes *i* and *j* in the above expression must to be replaced by sequences of indexes (*i*_1_, …, *i*_*n*_), (*j*_1_, …, *j*_*k*_), respectively [20].

The evaluation of MI for a set of features X and a set of receptors R requires a single data set of chemical compounds and corresponding activity labels Y for all receptors. This makes technically impossible the application of MI for a determination of the most informative subset of features with respect to various receptors because there usually does not exist a representative data set where each compound has proven activity or inactivity given arbitrary r∈R.

To overcome this problem, the calculation of MI(X;Y) was replaced by the computation of individual factors $\text{MI}(X;Y_{i})$. These partial results are gathered into final form by averaging:

$$\begin{array}{c} {\text{AIC}}_{Y}\left(X\right)=\frac{1}{K}\sum_{i=1}^{K}\frac{\text{MI}(X;Y_{i})}{\text{SE}(Y_{i})}. \end{array}$$

The normalization by the entropy of *Y*_*i*_ ensures that every factor describes the percentage of joint information instead of the absolute amount of information. In particular:

$$\begin{array}{c} 0\leq {\text{AIC}}_{Y}\left(X\right)\leq 1. \end{array}$$

## Results and Discussion

The experiments concerned the application of the AIC-Max algorithm for the selection of the most significant bits for ligands acting on five closely related biological receptors: 5-HT_2*A*_, 5-HT_2*B*_, 5-HT_2*C*_, 5-HT_5*A*_, 5-HT_6_. Among all fingerprints generated in the PaDEL software, only non-hashed fingerprints were considered: EState, MACCS, PubChem and Substructure (possessing 1434 bits in total) to ensure the structural analysis of selected bits (Table 3). Although hashed representations can be more efficient for classification purposes, their coordinates do not have a straightforward meaning. Therefore, they were not incorporated into the selection process. Moreover, the longest fingerprint (KRFP), although it was non-hashed, was skipped because a high number of bits results in a rapid increase of the computational time required by the feature selection process. Clearly, some of the chemical patterns can be duplicated while concatenating the above four fingerprints together. Nevertheless, since the repeated addition of the same feature does not increase the value of AIC, there is no risk that the algorithm will pick two identical (or even very similar) bits for final representation.

**Table 3:** Fingerprints generated in PaDEL software [18].

Fingerprint	Abbreviation	Hashed	Length
EState fingerprint [24]	estate	NO	79
MACCS fingerprint [25]	maccs	NO	166
PubChem fingerprint [18]	pubchem	NO	881
Substructure fingerprint [18]	substructure	NO	308
Klekota Roth fingerprint [9]	KRFP	NO	4860
Fingerprint [26]	fingerprint	YES	1024
Extended fingerprint [18]	extended	YES	1024
Graph-only fingerprint [18]	graph only	YES	1024

All ligands were extracted from ChEMBL database version 20 (February 2015) [27]. Ligands with an inhibition constant (*K*_*i*_) less than or equal to 100 nM were considered active; ligands with *K*_*i*_ higher than 1000 nM were used as inactives. Putative inactive compounds were randomly selected from the ZINC database [28] in a ratio of 9 inactives per 1 active (Table 4) [29].

**Table 4:** The summary of datasets used in the selection process.

Receptor	Actives	Inactives	ZINC
5-HT_2*A*_	2060	1081	18540
5-HT_2*B*_	428	341	3852
5-HT_2*C*_	1303	1050	11727
5-HT_5*A*_	69	146	621
5-HT_6_	1626	426	14634
5-HT_1*A*_	4427	1230	39843

To evaluate the significance of the selected features, a 10-fold cross-validation was performed [30]. In this approach, a dataset is randomly partitioned into 10 equally sized subsets. Then, a single subset is retained as test data while the remaining 9 subsets are used in training. This process is repeated 10 times—each of 10 subsamples is used exactly once as the test data, and the results are averaged. The AIC-Max algorithm was run on a training data set (including actives, inactives and putative inactives), and the evaluation of selected features was reported for a test set. The score was measured by the normalized mutual information Eq (2) between the constructed representation and the true activity labels for each of the receptors.

**Fig 3:**
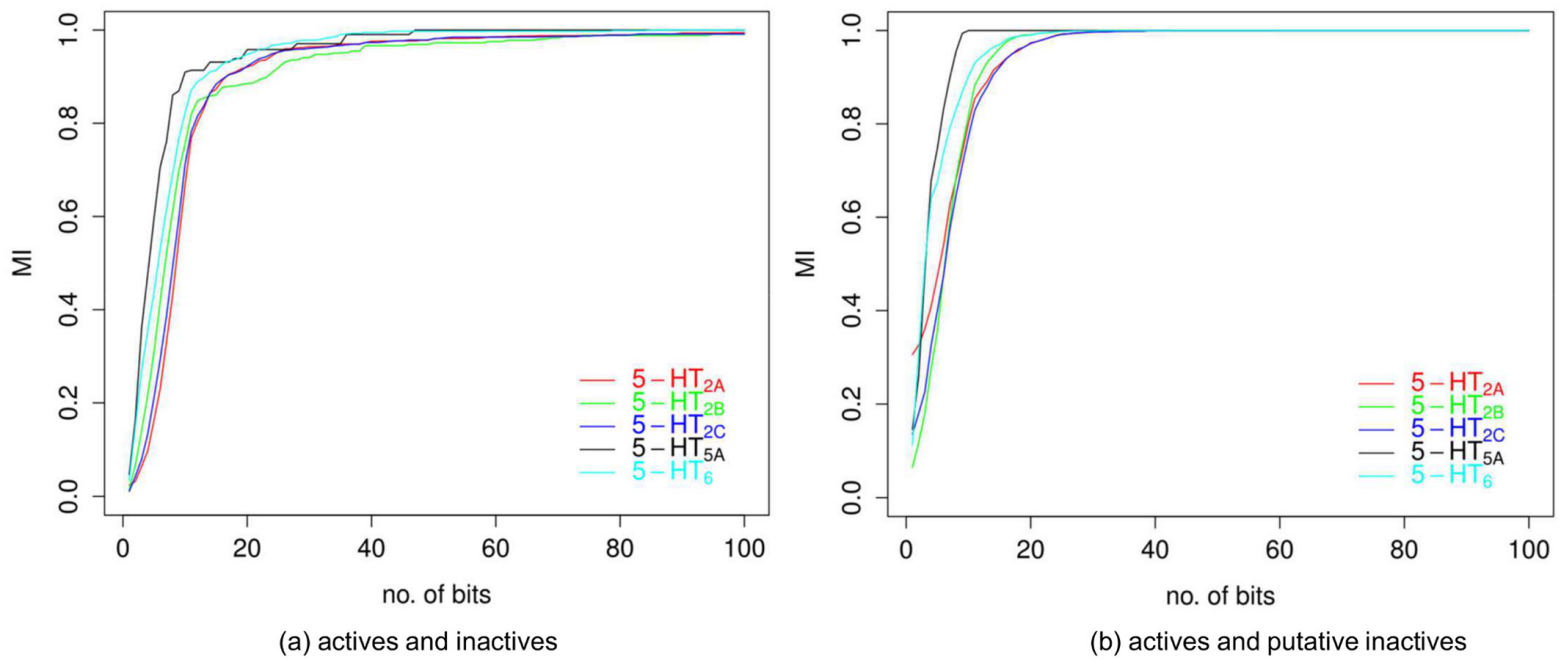
The relationship between the number of bits selected by the AIC-Max algorithm and associated information of activity. The information score was measured by the normalized mutual information calculated for constructed representations for every receptor averaged over all folds reported on a test set.

Information stored in a reduced fingerprint grows gradually with the increase in the number of features selected by AIC-Max algorithm (Fig 3). The level of 90% was rapidly attained by a representation containing approximately 20 bits for both datasets containing true inactives and compounds selected from ZINC. Nevertheless, to distinguish almost all considered active compounds from inactives, a set of 100 bits is required (more than 99% of information), while for putative inactives, only 30 bits suffice (close to 100% of information). This outcome is due to two particular reasons: the close structural similarity between actives and true inactives and the small amount of compounds with confirmed inactivity (Table 4).

Because the AIC-Max algorithm returned slightly different subsets of bits in each fold, the algorithm was additionally applied to the entire dataset to obtain a single set of features. The reduced fingerprint (see S1 File for details) contained features that are crucial in ligand-protein interaction for serotonin receptors: a positively polarizable nitrogen atom and an aromatic system [31]. Moreover, the bit encoding the tertiary nitrogen atom is the most desirable in the reduction and hybridization process. Polarizable nitrogen atoms are encoded by several bits listed in the top-scored instances. The same situation can also be observed for the aromatic system, which appears three times out of the 10 most desirable bits. Amide and sulfonamide moieties (and their subelements) are another popular patterns present in universal fingerprint, which reflect actual trends in medicinal chemistry [32–36].

The quality of the bits chosen by the AIC-Max algorithm was verified in a classification experiment conducted for the 5 underlying serotonin receptor ligands. As a classification method, a random forests technique [19] implemented in *randomForest R package* was used because it is known to be one of the state-of-the-art approaches in activity prediction [6]. The accuracy of classification was evaluated via Matthews Correlation Coefficient (*MCC*), the well-known validation measure, especially for imbalanced datasets. This measure is defined as [37]:

$$\begin{array}{c} MCC=\frac{TP\cdot TN-FP\cdot FN}{\sqrt{\left(TP+FP)(TP+FN)(TN+FP)(TN+FN\right)}}, \end{array}$$

where *TP* stands for the number of true positives (actives labeled as actives), *TN*—true negatives, *FP*—false positives (inactives labeled as actives) and *FN*—false negatives. *MCC* takes values from -1 to +1; The number +1 represents perfect prediction while 0 represents random prediction and − 1 represents an inverse prediction.

The experiment also assumed a 10-fold cross-validation procedure; a training set was used for a selection of bits and training of a classifier which was then evaluated on a test set. In each fold the AIC-Max algorithm was run for a merged set of actives, inactives and putative inactives to enforce generality of representation. On the other hand, the classifier was trained and tested separately on compounds of proven activity and on datasets containing active and putative inactive compounds.

**Fig 4:**
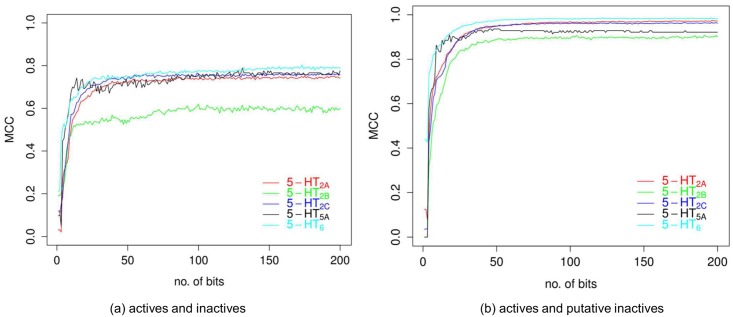
Classification performance. The relationship between the number of bits selected by AIC-Max algorithm and associated MCC score for every receptor averaged over all folds reported on a test set.

The addition of new features leads to the statistical improvement of the classification results (Fig 4). The highest increase was reported for representations including less than 20 bits. For a higher number of features, the difference in classification accuracy changes slightly. Because the gain in MCC value for representations containing more than 100 bits is negligible; then, longer representations were not taken into further consideration.

**Fig 5:**
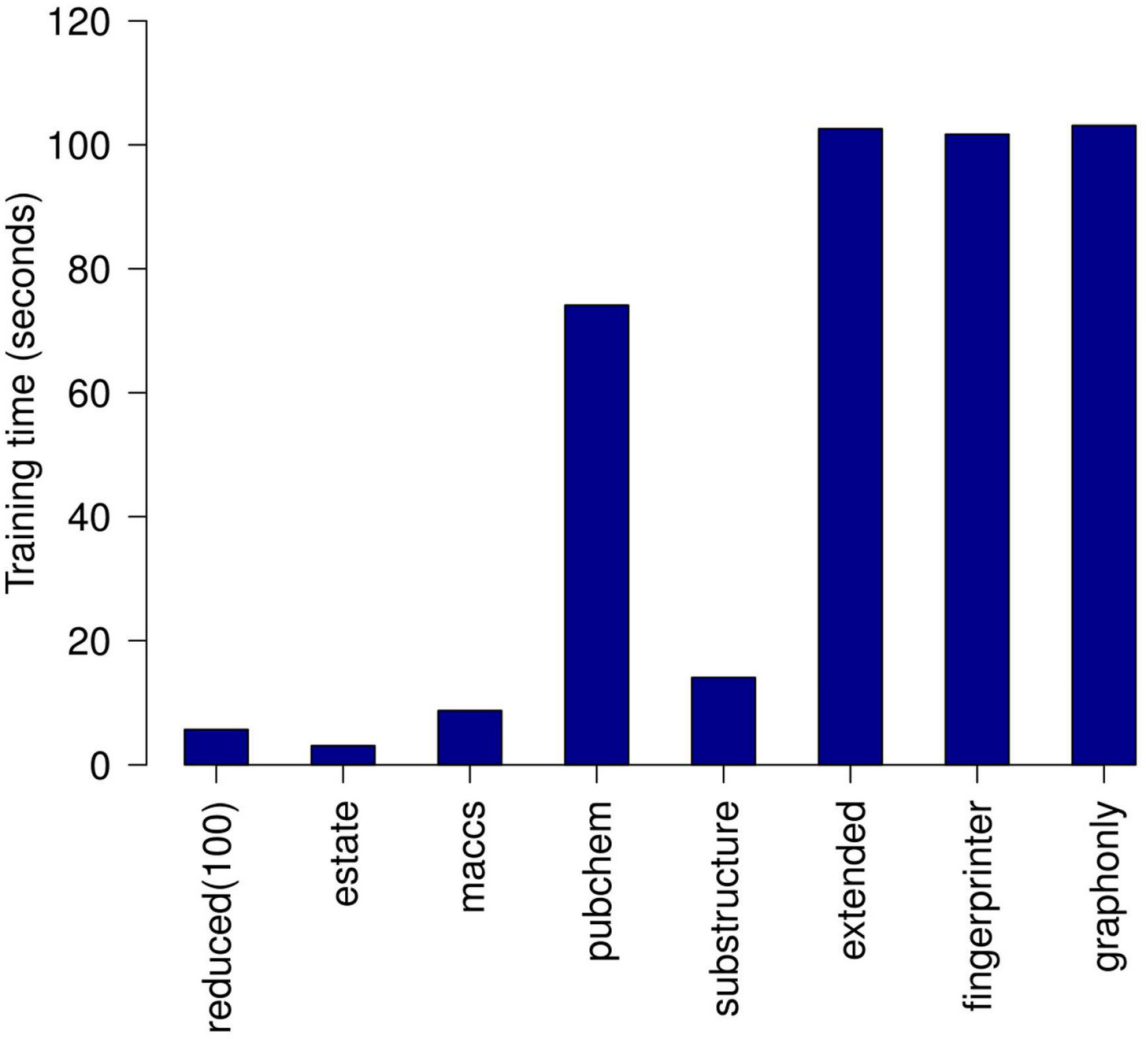
Classification times. Mean training times of a random forest classifier for various fingerprint representations averaged over all data sets of active and inactive compounds.

The classification performance of the representation created for 25, 50 and 100 bits was then compared with original (raw) fingerprints (Tables 5 and 6). The reduced representations including 100 as well as 50 bits outperformed existing fingerprints on all receptors when putative inactive compounds were used. This case is considered the most important one because it reflects virtual screening campaigns [29]. In the case of true inactives, the average MCC score of representation including 100 coordinates was comparable to the best performing hashed fingerprints. Moreover, the time required for training a classifier was approximately 17 times lower when a reduced 100-bits representation was used instead of any of the hashed fingerprints (Fig 5).

**Table 5:** Classification performance on a dataset containing actives and inactives.

fingerprint	5-HT_2*A*_	5-HT_2*B*_	5-HT_2*C*_	5-HT_5*A*_	5-HT_6_	mean
reduced(25)	0.679	0.521	0.708	0.698	0.737	0.669
reduced(50)	0.731	0.558	0.743	0.724	0.746	0.701
reduced(100)	0.736	**0.620**	0.761	0.759	0.778	**0.731**
estate	0.425	0.448	0.501	0.614	0.584	0.514
maccs	0.713	0.607	0.741	0.760	0.755	0.715
pubchem	0.730	0.545	0.739	**0.790**	0.739	0.709
substructure	0.500	0.483	0.551	0.647	0.595	0.555
KRFP	0.697	0.565	0.707	0.766	0.742	0.695
extended	**0.744**	0.596	**0.774**	0.736	0.803	0.730
fingerprinter	0.733	0.591	0.773	0.745	**0.806**	0.730
graphonly	0.703	0.559	0.716	0.788	0.774	0.708

**Table 6:** Classification performance on a dataset containing actives and putative inactives.

fingerprint	5-HT_2*A*_	5-HT_2*B*_	5-HT_2*C*_	5-HT_5*A*_	5-HT_6_	mean
reduced(25)	0.889	0.828	0.887	0.876	0.933	0.883
reduced(50)	0.939	0.878	0.939	**0.926**	0.966	0.929
reduced(100)	**0.959**	**0.885**	**0.952**	0.919	**0.971**	**0.937**
estate	0.604	0.503	0.563	0.725	0.844	0.648
maccs	0.936	0.877	0.932	0.894	0.970	0.922
pubchem	0.931	0.839	0.916	0.886	0.967	0.908
substructure	0.820	0.660	0.743	0.783	0.906	0.782
KRFP	0.932	0.841	0.925	0.862	0.965	0.905
extended	0.936	0.858	0.920	0.884	0.967	0.913
fingerprinter	0.932	0.852	0.918	0.868	0.966	0.907
graphonly	0.916	0.823	0.896	0.888	0.954	0.895

Finally, the generalization ability of created representation for another serotonin receptor was examined. A classification experiment was conducted on 5-HT_1*A*_ receptor ligands assuming reduced representation selected for five base receptors. Surprisingly, the extended fingerprint achieved perfect precision for the first dataset including compounds with proven activity or inactivity (Table 7). Although the reduced representation gave a significantly lower result, MCC = 0.663, it performed better than any of non-hashed fingerprints. In the case of putative inactives, the performance of constructed representation was slightly better than the MACCS and Extended fingerprints.

**Table 7:** Classification performance on a dataset containing active and inactive compounds of 5-HT_1*A*_ receptor (middle column) as well as actives and putative inactives (last column). The reduced representation was constructed from four non-hashed fingerprints based on five biological targets (first 3 rows). The reduced representation from all fingerprints (except KRFP) was also evaluated (last row).

fingerprint	inactives	ZINC
reduced(25)	0.553	0.893
reduced(50)	0.632	0.950
reduced(100)	0.663	**0.963**
estate	0.250	0.566
maccs	0.630	0.961
pubchem	0.659	0.948
substructure	0.332	0.886
KRFP	0.650	0.958
extended	**1.000**	0.960
fingerprinter	0.713	0.957
graphonly	0.627	0.933
reduced (100) formed from all fingerprints	0.998	0.961

To complement the study and investigate deeper the discriminative power of Extended fingerprint, we also considered a representation created from all fingerprints (Table 3) except KRFP including hashed ones. The results (Table 7) showed that the enhancement by bits from the hashed fingerprints significantly improved the statistics and gave almost ideal separation of actives from inactives.

Analogue experiments were conducted also for four another families of biological targets: carbonic anhydrases, cathepsins, histamine receptors and kinases (see S1 File).

## Conclusion

The paper introduced the AIC-Max algorithm as a method for fingerprint reduction and hybridization. The algorithm iteratively picks features uncorrelated among themselves to maximize AIC—a modified version of mutual information. In the present study, the algorithm was applied for constructing an essential representation of ligands of five families of closely related tergets. Such a representation can compete with raw fingerprints in classification experiments with significant CPU time reduction. The obtained results confirm that existing fingerprints contain much irrelevant information that may negatively influence on screening performance. The conducted experiments indicate that the generation and application of reduced and hybridized fingerprint allow rapid and effective calculations. The power of the methodology is underlined by the presence in universal representation bits that encode the most important structural features for serotonin receptor ligands: a polarizable nitrogen atom and the aromatic system.

## Supporting Information

S1 File The additional file, which can be retrieved from: http://www.ii.uj.edu.pl/~smieja/aic, contains the full list of 100 most informative bits selected from four non hashed fingerprints for five GPCRS receptors (Table A in S1 File) and the results of experiments conduced for the families of carbonic anhydrases (Tables B, F, J and K in S1 File), cathepsins (Tables C, G, L and M in S1 File, histamine receptors (Tables D, H, N and O in S1 File) and kinases (Tables E, I, Q and P in S1 File). (PDF)
