# Supplementary material for: Average Information Content Maximization—A New Approach for Fingerprint Hybridization and Reduction
Source: PLoS One. 2016 Jan 19;11(1):e0146666. doi: 10.1371/journal.pone.0146666 (PMC4718645; doi:10.1371/journal.pone.0146666)
Supplement: S1 File — (PDF) [file pone.0146666.s001.pdf]

Supporting Information

The supplementary information contains:

I. top 100 the most informative bits selected for the 5-HT$_{2A}$, 5-HT$_{2B}$, 5-HT$_{2C}$, 5-HT$_{5A}$ and 5-HT$_{6}$ receptors considered in the paper,
II. the results of the additional experiments concerning the application of the AIC-MAX algorithm for the selection of 100 the most significant bits for the families of cathepsin, carbonic anhydrases, histamine receptors and kinases.

I. Bits selected for 5-HT$_{2A}$, 5-HT$_{2B}$, 5-HT$_{2C}$, 5-HT$_{5A}$ and 5-HT$_{6}$ receptors.

Table A. The first 100 the most informative bits selected for the 5-HT$_{2A}$, 5-HT$_{2B}$, 5-HT$_{2C}$, 5-HT$_{5A}$ and 5-HT$_{6}$ receptors.

Rank	Fingerprint	Bit number	Original description
1	substructure	26	tertiary aliph amine
2	pubchem	759	Cc1c(Cl)cccc1
3	maccs	58	contains sulfur
4	pubchem	444	C(-Cl)(=O)
5	pubchem	529	Cl-C-C-Cl
6	pubchem	187	>= 2 saturated or aromatic nitrogen-containing ring size 6
7	maccs	105	('*@*(@*)@*',0), # A$A($A)$A
8	pubchem	539	N=C-C-[#1]
9	pubchem	146	>= 1 saturated or aromatic heteroatom-containing ring size 5
10	maccs	133	('*@*!@[#7]',0), # A$A!N
11	pubchem	406	O(~C)(~H)
12	pubchem	556	C=C-C-C
13	estate	36	secondary aliphatic oxygen
14	pubchem	540	C-N-C-[#1]
15	substructure	88	carboxylic acid derivative
16	pubchem	644	C-C=N-N-C
17	substructure	23	primary amine
18	maccs	86	('[C;H2,H3][!#6;!#1][C;H2,H3]',0), # CH2QCH2
19	substructure	287	conjugated double bond
20	pubchem	193	>= 3 saturated or aromatic carbon-only ring size 6
21	maccs	110	('[#7]~[#6]~[#8]',0), # NCO
22	maccs	134	contains halogen
23	pubchem	612	N-C-O-C-C
24	maccs	93	methyl group

25	maccs	92	92:('[#8]~[#6](~[#7])~[#6]',0), # OC(N)C
26	maccs	129	'[$(*~[CH2]~*~*~[CH2]~*),$([R]1@[CH2]@[R]@[R]@[CH2;R]1),$(*~[CH2]~[R]1@[R]@[CH2;R]1)]',0), # ACH2AACH2A
27	pubchem	698	O-C-C-C-C-C-C-C
28	estate	13	tertiary aliphatic carbon
29	pubchem	713	Cc1ccc(C)cc1
30	maccs	136	('[#8]=*',1), # O=A>1
31	estate	28	double bonded nitrogen
32	pubchem	681	O-C-C-C-C-O
33	pubchem	393	N(~C)(~H)
34	maccs	154	carbonyl
35	maccs	159	no of oxygen > 1
36	maccs	83	('[!#6;!#1]1~*~*~*~*~1',0), # QAAAA@1
37	pubchem	20	>= 4 O
38	pubchem	570	C:C-C-C
39	substructure	1	primary carbon
40	pubchem	440	C(-C)(-O)(=O)
41	maccs	107	('[F,Cl,Br,I]~*(~*)~*',0), # XA(A)A
42	estate	24	secondary aliphatic amine
43	pubchem	432	C(-C)(-C)(=O)
44	pubchem	421	C=S
45	pubchem	378	C(~N)(:C)(:N)
46	maccs	150	('*!@*@*!@*',0), # A!A$A!A
47	maccs	144	('*!:*:*!:*',0), # Anot%A%Anot%A
48	pubchem	697	C-C-C-C-C-C(C)-C
49	pubchem	822	CC1C(Cl)CCCC1
50	pubchem	452	C(-O)(=O)
51	pubchem	443	C(-C)(=O)
52	pubchem	181	>= 1 saturated or aromatic heteroatom-containing ring size 6
53	substructure	181	hetero nonbasic nitrogen
54	maccs	117	('[#7]~*~[#8]',0), # NAO
55	pubchem	147	>= 1 unsaturated non-aromatic carbon-only ring size 5
56	maccs	62	('*@*!@*@*',0), # A$!A$A
57	estate	29	aromatic secondary nitrogen
58	maccs	135	('[#7]!:*:*',0), # Nnot%A%A
59	pubchem	394	N(~C)(~H)(~N)
60	pubchem	454	N(-C)(=O)
61	maccs	77	('[#7]~*~[#7]',0), # NAN

62	maccs	91	('[$([!#6;!#1;!H0]~*~*~*~[CH2]~*),$([!#6;!#1;!H0;R]1@[R]@[R]@[R]@[CH2;R]1),$([!#6;!#1;!H0]~[R]1@[R]@[R]@[CH2;R]1),$([!#6;!#1;!H0]~*~[R]1@[R]@[CH2;R]1)]',0), # QHAAACH2A
63	pubchem	182	>= 1 unsaturated non-aromatic carbon-only ring size
64	pubchem	157	>= 2 aromatic rings
65	maccs	124	('[!#6;!#1]~[!#6;!#1]',0), # QQ
66	maccs	80	('[#7]~*~*~*~[#7]',0), # NAAAN
67	pubchem	534	O=C-C-N
68	pubchem	376	C(~N)(:C)
69	maccs	90	('[$([!#6;!#1;!H0]~*~*~[CH2]~*),$([!#6;!#1;!H0;R]1@[R]@[R]@[CH2;R]1),$([!#6;!#1;!H0]~[R]1@[R]@[CH2;R]1)]',0), # QHAACH2A
70	maccs	104	('[!#6;!#1;!H0]~*~[CH2]~*',0), # QHACH2A
71	pubchem	646	O=C-N-C-[#1]
72	pubchem	446	C(-H)(=C)
73	substructure	184	heteroaromatic
74	estate	35	double bonded oxygen
75	pubchem	575	O-C-C:C-O
76	maccs	96	5-membered ring
77	maccs	116	('[$([CH3]~*~*~[CH2]~*),$([CH3]~*1~*~[CH2]1)]',0), # CH3AACH2A
78	maccs	119	double bonded nitrogen
79	estate	30	tertiary aliph amine
80	maccs	127	('*@*!@[#8]',1), # A$A!O > 1 (&...) Spec Incomplete
81	maccs	16	('[!#6;!#1]1~*~*~1',0), # QAA@1
82	pubchem	339	(~C)(~C)(~H)(~O)
83	maccs	157	single bonded oxygen
84	maccs	125	no of aromatic rings > 1
85	maccs	128	('[$(*~[CH2]~*~*~*~[CH2]~*),$([R]1@[CH2;R]@[R]@[R]@[R]@[CH2;R]1),$(*~[CH2]~[R]1@[R]@[R]@[CH2;R]1),$(*~[CH2]~*~[R]1@[R]@[CH2;R]1)]',0), # ACH2AAACH2A
86	pubchem	594	C-O-C-C=C
87	pubchem	647	O=C-N-C-N
88	pubchem	386	C(:C)(:C)(:N)
89	maccs	112	('*~*(~*)(~*)~*',0), # AA(A)(A)A
90	maccs	151	secondary amine
91	maccs	143	('*@*!@[#8]',0), # A$A!O

92	pubchem	287	C-F
93	maccs	79	('[#7]~*~*~[#7]',0), # NAAN
94	maccs	95	('[#7]~*~*~[#8]',0), # NAAO
95	maccs	138	('[!#6;!#1]~[CH2]~*',1), # QCH2A>1 (&...) Spec Incomplete
96	maccs	98	('[!#6;!#1]1~*~*~*~*~*~1',0), # QAAAAA@1
97	pubchem	419	C#N
98	pubchem	367	C(~H)(~O)(~O)
99	maccs	102	('[!#6;!#1]~[#8]',0), # QO
100	pubchem	258	>= 2 hetero-aromatic rings

II. The additional experiment focused on the application of AIC-MAX algorithm for the selection of 100 most informative bits for the families of cathepsin, carbonic anhydrases, histaminereceptorsand kinases. For the first case the cathepsins B, K, L and S were considered (**Table B**), for the second family carbonic anhydrases I (CAI), II (CAII), IX (CAIX) and XII (CAXII) were assumed (**Table C**), for histamine H_1, H_2 and H_3 were taken(**Table D**) while for kinases ABL, LCK and SRC were used (**Table E**). All those data were prepared in the same manner as a data for serotonin receptor family described in the main text.

Table B. The summary of carbonic anhydrases inhibitors datasets.

**Receptor**	**Actives**	**Inactives**	**ZINC**
CAI	956	1777	8605
CAII	2445	978	21997
CAIX	1189	475	10693
CAXII	920	280	8277

Table C. The summary of cathepsins inhibitors datasets.

**Receptor**	**Actives**	**Inactives**	**ZINC**
CatB	246	1078	2206
CatK	1039	525	9334
CatS	944	379	8488
CatL	371	1132	3332

Table D. The summary of histamine receptorsdatasets.

**Receptor**	**Actives**	**Inactives**	**ZINC**
H_1	636	546	5715
H_2	12	95	60
H_3	2706	313	24345

Table E. The summary of kinases datasets.

Receptor	Actives	Inactives	ZINC
ABL	409	582	3689
LCK	857	1407	7721
SRC	943	2174	8495

The lists of best performing bits for particular families are presented in **Tables F, G, H and I,** respectively. It was examined that the representations consisting of the first 100 bits contain more than 99% of information needed to distinguish actives from inactives for all families.

Table F. The first 100 the most informative bits selected for the ligands from the carbonic anhydrases family (CA I, CA II, CA IX and CA XII).

Rank	Fingerprint	Bit number
1	estate	21
2	maccs	73
3	pubchem	391
4	maccs	117
5	maccs	105
6	maccs	77
7	pubchem	35
8	estate	9
9	substructure	1
10	maccs	95
11	pubchem	690
12	maccs	55
13	maccs	149
14	maccs	125
15	maccs	121
16	maccs	144
17	maccs	84
18	pubchem	536
19	maccs	131
20	substructure	214
21	maccs	153
22	maccs	154
23	maccs	69
24	pubchem	258
25	maccs	127
26	maccs	147
27	pubchem	378

28	maccs	160
29	pubchem	419
30	pubchem	375
31	pubchem	346
32	pubchem	394
33	pubchem	357
34	pubchem	614
35	maccs	51
36	maccs	33
37	pubchem	366
38	maccs	97
39	pubchem	496
40	maccs	150
41	pubchem	306
42	pubchem	657
43	maccs	83
44	pubchem	460
45	pubchem	421
46	maccs	100
47	pubchem	17
48	pubchem	484
49	pubchem	186
50	maccs	98
51	maccs	110
52	maccs	32
53	maccs	47
54	maccs	140
55	maccs	137
56	estate	7
57	maccs	155
58	pubchem	431
59	substructure	275
60	estate	30
61	pubchem	21
62	maccs	143
63	pubchem	452
64	pubchem	376
65	maccs	92
66	substructure	143
67	substructure	88
68	pubchem	503
69	pubchem	415
70	pubchem	3
71	estate	24
72	pubchem	444
73	pubchem	549

74	pubchem	422
75	pubchem	466
76	maccs	133
77	estate	53
78	pubchem	187
79	substructure	209
80	substructure	287
81	pubchem	529
82	pubchem	438
83	maccs	58
84	maccs	96
85	pubchem	646
86	maccs	61
87	pubchem	608
88	estate	16
89	maccs	158
90	pubchem	458
91	pubchem	144
92	pubchem	620
93	maccs	94
94	maccs	122
95	maccs	152
96	maccs	60
97	maccs	157
98	pubchem	450
99	substructure	297
100	pubchem	629

Table G. The first 100 the most informative bits selected for the ligands from the cathepsin inhibitors family (B, K, L and S).

Rank	Fingerprint	Bit number
1	pubchem	603
2	maccs	41
3	pubchem	4
4	substructure	153
5	maccs	85
6	maccs	80
7	pubchem	693
8	maccs	112
9	maccs	82
10	maccs	132
11	pubchem	336
12	pubchem	392
13	maccs	135

14	estate	15
15	pubchem	17
16	substructure	143
17	pubchem	537
18	pubchem	613
19	maccs	54
20	pubchem	698
21	pubchem	420
22	estate	19
23	maccs	124
24	substructure	133
25	substructure	3
26	maccs	129
27	substructure	287
28	pubchem	339
29	maccs	115
30	pubchem	697
31	pubchem	570
32	maccs	106
33	pubchem	705
34	pubchem	612
35	estate	26
36	pubchem	419
37	maccs	122
38	maccs	16
39	pubchem	634
40	maccs	23
41	maccs	74
42	maccs	100
43	maccs	131
44	pubchem	594
45	maccs	97
46	pubchem	453
47	pubchem	430
48	maccs	148
49	maccs	133
50	maccs	91
51	pubchem	347
52	pubchem	465
53	pubchem	193
54	maccs	150
55	maccs	90
56	estate	30
57	pubchem	147
58	maccs	86
59	pubchem	567

60	maccs	95
61	maccs	126
62	substructure	100
63	maccs	75
64	pubchem	685
65	maccs	130
66	maccs	140
67	pubchem	378
68	maccs	66
69	pubchem	386
70	maccs	104
71	substructure	49
72	maccs	120
73	maccs	152
74	maccs	128
75	estate	18
76	pubchem	443
77	pubchem	187
78	maccs	79
79	pubchem	550
80	pubchem	615
81	pubchem	556
82	pubchem	560
83	maccs	94
84	pubchem	21
85	pubchem	554
86	maccs	138
87	maccs	146
88	maccs	98
89	maccs	123
90	pubchem	496
91	maccs	144
92	pubchem	598
93	maccs	149
94	maccs	105
95	pubchem	438
96	pubchem	647
97	pubchem	673
98	pubchem	629
99	maccs	107
100	maccs	77

Table H. The first 100 the most informative bits selected for the ligands from the histamine receptors family (H_1, $H_2$and H_3).

Rank	Fingerprint	Bit number
1	pubchem	183
2	substructure	17
3	maccs	144
4	pubchem	668
5	maccs	131
6	pubchem	358
7	pubchem	673
8	substructure	26
9	maccs	106
10	maccs	95
11	pubchem	347
12	pubchem	393
13	maccs	80
14	pubchem	181
15	pubchem	693
16	pubchem	432
17	pubchem	529
18	pubchem	256
19	pubchem	150
20	pubchem	486
21	maccs	110
22	pubchem	190
23	pubchem	524
24	pubchem	180
25	pubchem	705
26	substructure	23
27	pubchem	646
28	pubchem	149
29	pubchem	647
30	substructure	287
31	maccs	125
32	pubchem	185
33	pubchem	294
34	maccs	62
35	maccs	150
36	substructure	3
37	maccs	126
38	pubchem	612
39	estate	24
40	pubchem	699
41	maccs	135
42	maccs	116

43	substructure	18
44	maccs	149
45	maccs	134
46	pubchem	629
47	pubchem	182
48	maccs	105
49	pubchem	570
50	pubchem	258
51	pubchem	193
52	maccs	77
53	pubchem	257
54	substructure	1
55	substructure	184
56	pubchem	621
57	pubchem	184
58	pubchem	448
59	pubchem	339
60	maccs	79
61	estate	13
62	maccs	151
63	pubchem	300
64	pubchem	644
65	maccs	93
66	pubchem	697
67	maccs	118
68	maccs	90
69	pubchem	394
70	pubchem	539
71	pubchem	619
72	maccs	86
73	maccs	133
74	maccs	94
75	maccs	120
76	pubchem	633
77	pubchem	388
78	pubchem	397
79	maccs	117
80	pubchem	147
81	maccs	97
82	substructure	88
83	maccs	91
84	pubchem	421
85	pubchem	594
86	pubchem	452
87	maccs	81
88	pubchem	698

89	maccs	87
90	maccs	108
91	maccs	139
92	estate	30
93	pubchem	374
94	maccs	75
95	pubchem	639
96	maccs	109
97	pubchem	146
98	maccs	104
99	maccs	154
100	pubchem	503

Table I. The first 100 the most informative bits selected for the ligands from the kinases family (ABL, LCK and SRC).

**Rank**	**Fingerprint**	**Bit number**
1	pubchem	183
2	pubchem	622
3	pubchem	190
4	pubchem	485
5	pubchem	149
6	pubchem	713
7	substructure	23
8	pubchem	432
9	maccs	131
10	pubchem	699
11	maccs	107
12	pubchem	685
13	maccs	134
14	maccs	99
15	pubchem	373
16	pubchem	378
17	pubchem	436
18	maccs	100
19	pubchem	580
20	pubchem	637
21	pubchem	698
22	pubchem	542
23	pubchem	256
24	maccs	62
25	pubchem	185
26	pubchem	717
27	maccs	85
28	pubchem	184

29	pubchem	150
30	maccs	86
31	pubchem	759
32	pubchem	673
33	pubchem	780
34	pubchem	392
35	maccs	149
36	maccs	94
37	pubchem	554
38	maccs	75
39	pubchem	452
40	maccs	119
41	pubchem	646
42	pubchem	382
43	pubchem	602
44	substructure	1
45	pubchem	257
46	maccs	87
47	estate	30
48	estate	11
49	pubchem	383
50	pubchem	180
51	pubchem	258
52	pubchem	672
53	pubchem	697
54	pubchem	379
55	pubchem	439
56	pubchem	549
57	maccs	92
58	maccs	38
59	estate	13
60	substructure	137
61	pubchem	536
62	pubchem	822
63	pubchem	575
64	pubchem	494
65	pubchem	193
66	pubchem	594
67	pubchem	387
68	pubchem	358
69	pubchem	147
70	pubchem	675
71	maccs	83
72	pubchem	540
73	pubchem	182
74	pubchem	440

75	pubchem	597
76	pubchem	200
77	maccs	138
78	pubchem	598
79	pubchem	446
80	pubchem	146
81	pubchem	380
82	maccs	124
83	maccs	97
84	pubchem	181
85	pubchem	573
86	maccs	106
87	pubchem	529
88	maccs	96
89	pubchem	547
90	pubchem	444
91	pubchem	386
92	maccs	78
93	substructure	180
94	pubchem	578
95	maccs	125
96	maccs	155
97	pubchem	570
98	maccs	154
99	maccs	25
100	maccs	52

The usefulness of constructed fingerprints with 100 bits were further verified in the classification experiments and compared with the results obtained by original fingerprints (**Tables J, K, L, M, N, O, Q and P**). Performed tests indicate that reduced representations gave the best MCC score on average for all families except active and inactive compounds of kinases family. Its superiority over traditional fingerprints was the most evident in the case of cathepsins inhibitors and histamine families; it outperformed every fingerprint on all data sets except putative inactive compounds of Cathepsin B receptor (the difference between the results for reduced and maccs fingerprint was less than 0.001).

Table J. Classification performance on a dataset containing actives and inactives for carbonic anhydrases family.

**fingerprint**	**CAI**	**CAII**	**CAIX**	**CAXII**	**mean**
reduced(100)	0.837	0.826	0.859	0.931	0.863
estate	0.611	0.708	0.729	0.767	0.704
maccs	0.838	0.814	0.882	0.906	0.860

pubchem	0.828	0.807	0.898	0.906	0.860
substructure	0.644	0.745	0.760	0.782	0.733
extended	0.820	0.769	0.883	0.860	0.833
fingerprinter	0.803	0.785	0.869	0.910	0.842
graphonly	0.812	0.800	0.837	0.882	0.833

Table K. Classification performance on a dataset containing actives and putative inactives for carbonic anhydrases family.

Fingerprint	CAI	CAII	CAIX	CAXII	mean
reduced(100)	0.988	0.957	0.969	0.968	0.970
estate	0.964	0.931	0.945	0.967	0.952
maccs	0.970	0.966	0.976	0.957	0.967
pubchem	0.964	0.960	0.962	0.932	0.955
substructure	0.858	0.854	0.848	0.867	0.857
extended	0.928	0.944	0.957	0.938	0.942
fingerprinter	0.946	0.937	0.967	0.938	0.947
graphonly	0.964	0.947	0.953	0.926	0.947

Table L. Classification performance on a dataset containing actives and inactives for cathepsins family.

Fingerprint	CatB	CatK	CatS	CatL	mean
reduced(100)	0.977	0.973	0.976	0.939	0.966
estate	0.519	0.619	0.750	0.525	0.603
maccs	0.976	0.940	0.958	0.907	0.945
pubchem	0.929	0.956	0.939	0.874	0.925
substructure	0.718	0.878	0.876	0.789	0.815
extended	0.904	0.951	0.945	0.923	0.931
fingerprinter	0.904	0.945	0.952	0.923	0.931
graphonly	0.953	0.934	0.964	0.891	0.935

Table M. Classification performance on a dataset containing actives and putative inactives for cathepsins family.

Fingerprint	CatB	CatK	CatS	CatL	mean
reduced(100)	0.976	0.973	0.976	0.939	0.966
estate	0.519	0.619	0.750	0.525	0.603
maccs	0.977	0.940	0.958	0.907	0.945
pubchem	0.929	0.956	0.939	0.874	0.925
substructure	0.718	0.878	0.876	0.789	0.815
fingerprinter	0.904	0.945	0.952	0.923	0.931
extended	0.904	0.951	0.945	0.923	0.931

graphonly	0.953	0.934	0.964	0.891	0.935

Table N. Classification performance on a dataset containing actives and inactives for histamine receptors family.

Fingerprint	H_1	H_2	H_3	mean
reduced(100)	0.763	0.646	0.686	0.698
estate	0.518	0.410	0.237	0.388
maccs	0.763	0.643	0.686	0.697
pubchem	0.746	0.633	0.686	0.688
substructure	0.671	0.462	0.377	0.503
fingerprinter	0.723	0.627	0.655	0.668
extended	0.730	0.634	0.664	0.676
graphonly	0.688	0.576	0.619	0.628

Table O. Classification performance on a dataset containing actives and putative inactives for histamine receptors family.

Fingerprint	H_1	H_2	H_3	mean
reduced(100)	0.991	1.000	0.998	0.996
estate	0.698	0.574	0.623	0.632
maccs	0.920	0.921	0.967	0.936
pubchem	0.991	0.671	0.996	0.886
substructure	0.873	0.759	0.912	0.848
fingerprinter	0.881	0.945	0.979	0.935
extended	0.892	0.972	0.973	0.946
graphonly	0.841	0.919	0.951	0.904

Table Q. Classification performance on a dataset containing actives and inactives for kinases family.

Fingerprint	ABL	LCK	SRC	mean
reduced(100)	0.774	0.753	0.785	0.771
estate	0.569	0.594	0.685	0.616
maccs	0.730	0.743	0.735	0.736
pubchem	0.811	0.771	0.748	0.777
substructure	0.641	0.665	0.676	0.661
fingerprinter	0.814	0.714	0.769	0.766
extended	0.834	0.723	0.775	0.778
graphonly	0.788	0.684	0.732	0.735

Table P. Classification performance on a dataset containing actives and putative inactives for kinases family.

Fingerprint	ABL	LCK	SRC	mean
reduced(100)	0.986	1.000	1.000	0.995
estate	0.775	0.700	0.824	0.766
maccs	0.944	0.934	0.934	0.937
pubchem	0.986	0.993	0.994	0.991
substructure	0.855	0.700	0.811	0.789
fingerprinter	0.944	0.940	0.946	0.943
extended	0.929	0.940	0.946	0.938
graphonly	0.915	0.926	0.927	0.923
